# Proteomic Modulation in TGF-β-Treated Cholangiocytes Induced by Curcumin Nanoparticles

**DOI:** 10.3390/ijms241310481

**Published:** 2023-06-22

**Authors:** Elisa Ceccherini, Giovanni Signore, Lorena Tedeschi, Federico Vozzi, Nicoletta Di Giorgi, Elena Michelucci, Antonella Cecchettini, Silvia Rocchiccioli

**Affiliations:** 1 Institute of Clinical Physiology, National Research Council, 56124 Pisa, Italy; giovanni.signore@unipi.it (G.S.); tedeschi@ifc.cnr.it (L.T.); vozzi@ifc.cnr.it (F.V.); digiorgi@ifc.cnr.it (N.D.G.); elena.michelucci@pi.iccom.cnr.it (E.M.); antonella.cecchettini@unipi.it (A.C.); silvia.rocchiccioli@ifc.cnr.it (S.R.); 2 Biochemistry Unit, Department of Biology, University of Pisa, 56123 Pisa, Italy; 3 Institute of Chemistry of Organometallic Compounds, National Research Council, 56124 Pisa, Italy; 4 Department of Clinical and Experimental Medicine, University of Pisa, 56126 Pisa, Italy

**Keywords:** cholangiocytes, curcumin, nanoparticles, TGF-β, proteomics, LC-MS/MS

## Abstract

Curcumin is a natural polyphenol that exhibits a variety of beneficial effects on health, including anti-inflammatory, antioxidant, and hepato-protective properties. Due to its poor water solubility and membrane permeability, in the present study, we prepared and characterized a water-stable, freely dispersible nanoformulation of curcumin. Although the potential of curcumin nanoformulations in the hepatic field has been studied, there are no investigations on their effect in fibrotic pathological conditions involving cholangiocytes. Exploiting an in vitro model of transforming growth factor-β (TGF-β)-stimulated cholangiocytes, we applied the Sequential Window Acquisition of All Theoretical Mass Spectra (SWATH-MS)-based quantitative proteomic approaches to study the proteome modulation induced by curcumin nanoformulation. Our results confirmed the well-documented anti-inflammatory properties of this nutraceutic, highlighting the induction of programmed cell death as a mechanism to counteract the cellular damages induced by TGF-β. Moreover, curcumin nanoformulation positively influenced the expression of several proteins involved in TGF-β-mediated fibrosis. Given the crucial importance of deregulated cholangiocyte functions during cholangiopathies, our results provide the basis for a better understanding of the mechanisms associated with this pathology and could represent a rationale for the development of more targeted therapies.

## 1. Introduction

Curcumin (Cur) is a natural polyphenolic pigment that is extracted from turmeric with a broad safety profile [1]. This phytochemical has been used as an anti-inflammatory, anti-oxidant, anti-bacterial, anticarcinogenic, antifungal, and hepatoprotective agent [2]. Despite several benefits, Cur has limited water solubility and membrane permeability, and it is unstable at high temperatures when exposed to light and oxidative conditions [3]. After oral administration, it undergoes a fast first-pass metabolism and is quickly eliminated, thus reducing its bioavailability [4]. 

Nanoparticles (NPs) have a great impact on future medical treatments for many diseases. Compared to conventional therapies, drug-loaded NPs possess several advantages, including a prolonged circulation half-life and the elimination of side effects [5,6]. Over the last decade, scientific research has investigated the therapeutic potential of CurNPs in various pathological conditions, with the aim of overcoming their limitations and improving their pharmacologic profile. The hepato-protective effects of CurNPs have been partially elucidated, but relevant data from the literature also involve the anti-carcinogenic activity of CurNPs through their antioxidant, anti-inflammatory, and pro-apoptotic activities, as demonstrated in a mouse model of diethylnitrosamine-induced liver carcinoma [7]. Similarly, Mona and colleagues reported the hepato-protective effects of CurNPs treatment in HepG2 and Huh-7 cancer cells due to their viability reduction and apoptosis increase [8]. Recently, the anti-liver fibrosis activity of CurNPs has also been investigated. The phytochemical was able to maintain its hepatic function and architecture during fibrosis development in a CCl_4_-induced liver fibrosis mouse model by the direct inhibition of fibrosis-mediating proteins (e.g., the platelet-derived growth factor receptor beta, TIMP metallopeptidase inhibitor 1, Toll-like receptor 9 and TGF-β [9]. In another study, Ji and colleagues correlated the anti-fibrotic effect of CurNPs to their anti-inflammatory activity and to the up-regulation of hepatocyte growth factors and matrix metalloprotease-2 [10]. Although the potentiality of CurNPs in the hepatic field has been studied, there are no investigations on their effect in the fibrotic pathological conditions involving cholangiocytes. 

TGF-β is a cytokine that modulates several cellular processes, including proliferation, migration, invasion, angiogenesis, and immune responses [11]. During hepatic fibrosis, TGF-β promotes inflammation and regulates the hepatocyte’s behavior and activation of human stellate cells (HSCs), which could represent the main source of the extracellular matrix [12,13,14,15,16,17].

The main purpose of this work was to prepare and characterize a water stable, including the freely dispersible nanoformulation of curcumin, and to study the proteome modulation that it induces in a well-established model of in vitro TGF-β-stressed cholangiocytes [18].

## 2. Results

### 2.1. CurNPs Synthesis and Characterization 

First, we sought to set up an automated, robust method for the reproducible preparation of CurNPs. To this end, we adopted a nanoprecipitation approach using DMSO-dissolved curcumin and water as a non-solvent. A microfluidic-based protocol was implemented to allow for the preparation of CurNPs with a reproducible, uniform size and with the possibility to scale up the process. Different volume ratios between DMSO and water were tested, leading to CurNPs preparations 1:79 *v*/*v* (CurNP_a_), 1:158 *v*/*v* (CurNP_b_), 1:316 *v*/*v* (CurNP_c_), 1:625 *v*/*v* (CurNP_d_), and 1:1280 *v*/*v* (CurNP_e_). In all cases, these distributions were monodispersed with a relatively narrow distribution. The size and dispersion (evaluated by intensity distribution on DLS) obtained for the three preparations were 158 nm ± 3 nm (CurNP_a_), 120 nm ± 1 nm (CurNP_b_), 97 nm ± 2 nm (CurNP_c_), 95 nm ± 1 nm (CurNP_d_), and 97 nm ± 9 nm (CurNP_e_), indicating a slow, yet noticeably decreasing trend in CurNPs diameter with an increasing water/DMSO ratio. CurNPs were stabilized at approximately 97 nm for any water/DMSO ratio greater than 316:1, while larger sizes were obtained at lower ratios. The size distributions for the synthesized nanoparticles are reported in Figure S1. 

With the aim of using smaller, homogeneous, and highly concentrated CurNPs in subsequent experiments, attention was focused on CurNP_b_, CurNP_c_, and CurNP_d_. In fact, CurNP_e_ was too diluted to be of practical use, while CurNP_a_ was too polydisperse.

### 2.2. CurNPs Dispersion Characterization

The solutions of curcumin in DMSO maintained the same absorbance spectra over at least 48 h, while fluorescence decreased over time, reaching about 70% of the initial value in 48 h (Figure S2a–d). Curcumin in the DMSO was added to the culture medium or PBS to not produce real/stable solutions even if an amount of DMSO and serum was added. The absorbance and fluorescence decreased, and the discoloring of these solutions was evident (Figure S2e–h). 

CurNPs were produced instead of a more stable dispersion without any sign of precipitation over the hours. In order to exactly quantify the number of CurNPs per volume unit of the stock solution for each preparation, the absorbance of serial dilutions of CurNP_b_, CurNP_c,_ and CurNP_d_ in PBS was evaluated, and an “equivalent concentration” [µM] of curcumin was calculated from the standard curve of Curcumin in DMSO. CurNP_d_ (1:615) preparation was 13.1 µM, CurNP_c_ (1:316) was 55.6 µM and CurNP_b_ (1:158) 187.4 µM. CurNP_b_ was used as a stock solution to obtain the desired amount of CurNPs for the experiment with cells.

With the aim of assessing the behavior of CurNPs in the culture medium, the optical properties of their dispersions in the culture medium, with different amounts of the fetal bovine serum (FBS), were evaluated over time. In all the conditions tested, a reduction in the fluorescence emission was to be less than 40%; this is slightly higher than the reduction in fluorescence of curcumin in DMSO (30%) in the same interval (Figure S2i). The administration of CurNPs instead of “free” curcumin ensured that a “nominal” amount of curcumin in the medium could be reached (till 50 µM), and cells were not affected by the massive precipitation of aggregates. 

### 2.3. Sample Collection

**Figure 1:**
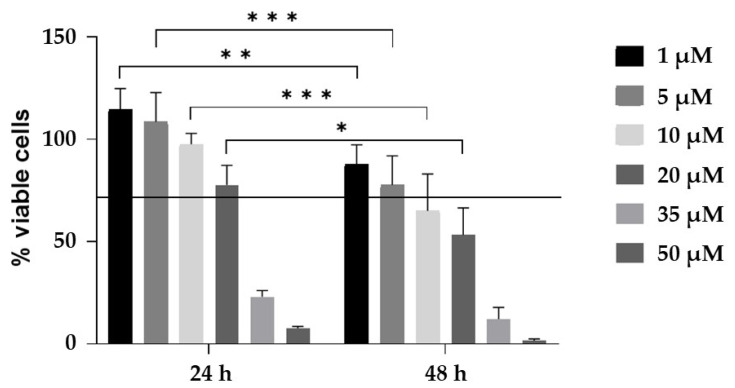
Cytotoxic effect of CurNPs-treated with cholangiocytes for 24 h and 48 h. Data were obtained from 3 independent experiments and are presented as the percentage of viable cells compared to control cells. Statistical analysis was performed with two-way ANOVA and Tukey’s multiple comparison test; * *p* ≤ 0.05, ** *p* ≤ 0.01, *** *p*≤ 0.001.

Primary human cholangiocytes were treated for 48h with TGF-β at a concentration of 10 ng/mL, in accordance with our previous study [18]. Another sample group was obtained by treating cholangiocytes with TGF-β 10 ng/mL for 24h before adding CurNPs 10 µM for 24 h. The CurNPs concentration and treatment time were determined by applying a viability cut-off greater than 70%, as shown in Figure 1. The control group was obtained by growing cholangiocytes in their specific culture medium with 1% FBS. At the end of the experiments, the cell pellets were collected for subsequent liquid chromatography-tandem mass spectrometry (LC-MS/MS) analysis.

### 2.4. IL-6 Dosage

**Figure 2:**
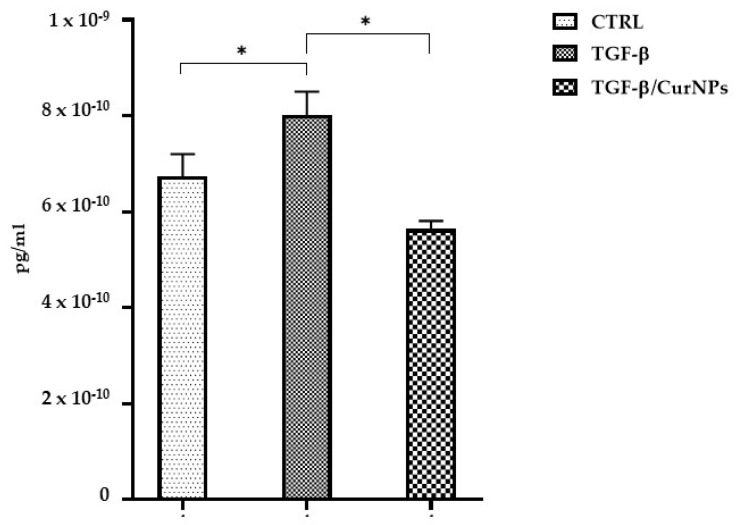
Levels of IL-6 in culture media. Data represent the mean of 3 independent experiments. Statistical analysis was performed with one-way ANOVA and Holm–Sidak’s multiple comparison test; * *p* ≤ 0.05.

The anti-inflammatory effect exerted by curcumin in multiple pathologic conditions, including liver diseases, is well known [19,20]. Hence, we evaluated interleukin-6 (IL-6) in the culture medium for each condition tested (Figure 2). In TGF-β-treated cholangiocytes, IL-6 levels significantly increased compared to the control cells; conversely, CurNPs seemed to reduce the levels of TGF- β-induced inflammation.

### 2.5. Hypothesis Free Proteomics and Bioinformatic Analysis

**Figure 3:**
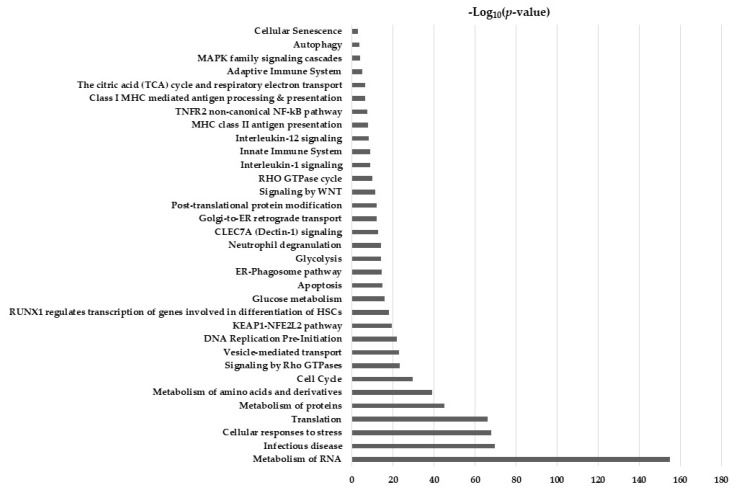
Biological functions of deregulated proteins analyzed using the David database; the significant ones (*p* ≤ 0.05) were reported.

Human cholangiocytes were subjected to protein extraction, reduction, alkylation, and digestion with trypsin, and the resulting peptide mixture was prepared for LC-MS/MS analysis, thus quantifying 2479 proteins. To obtain an overview of biological significance, these proteins were categorized according to their biological function using a David bioinformatic database. As reported in Figure 3, the enriched biological processes were primarily related to RNA metabolism, infections, proteins and amino acid metabolism, and cell cycle. Interestingly, the immune response and interleukin signaling were also identified.

To identify the dysregulated proteins during TGF-β and TGF-β/CurNPs treatments, a comparative analysis with the control cells and TGF-β-treated cholangiocytes was performed, respectively.

In TGF-β-treated cholangiocytes, a total of 28 proteins were significantly dysregulated with respect to the control cells greater than ±2.5-fold. In particular, data analysis showed 24 upregulated and 4 downregulated proteins following 48 h of treatment. Among them, fibronectin and dynamin-3 were the most deregulated proteins, with a fold-change (FC) of 295.85 and 0.01, respectively (Table 1).

The data analysis of TGF-β/CurNPs-treated cholangiocytes highlighted 10 significantly deregulated proteins compared to TGF-β-treated (48 h) cells. In particular, we showed eight upregulated and two downregulated proteins (Table 2). Among them, dynamin-3 and Ras-related protein Rab-15 were the most deregulated proteins, with a FC of 248.1 and 0.0014, respectively.

### 2.6. Comparative Analysis of TGF-β and TGF-β/CurNPs Dysregulated Proteins

**Figure 4:**
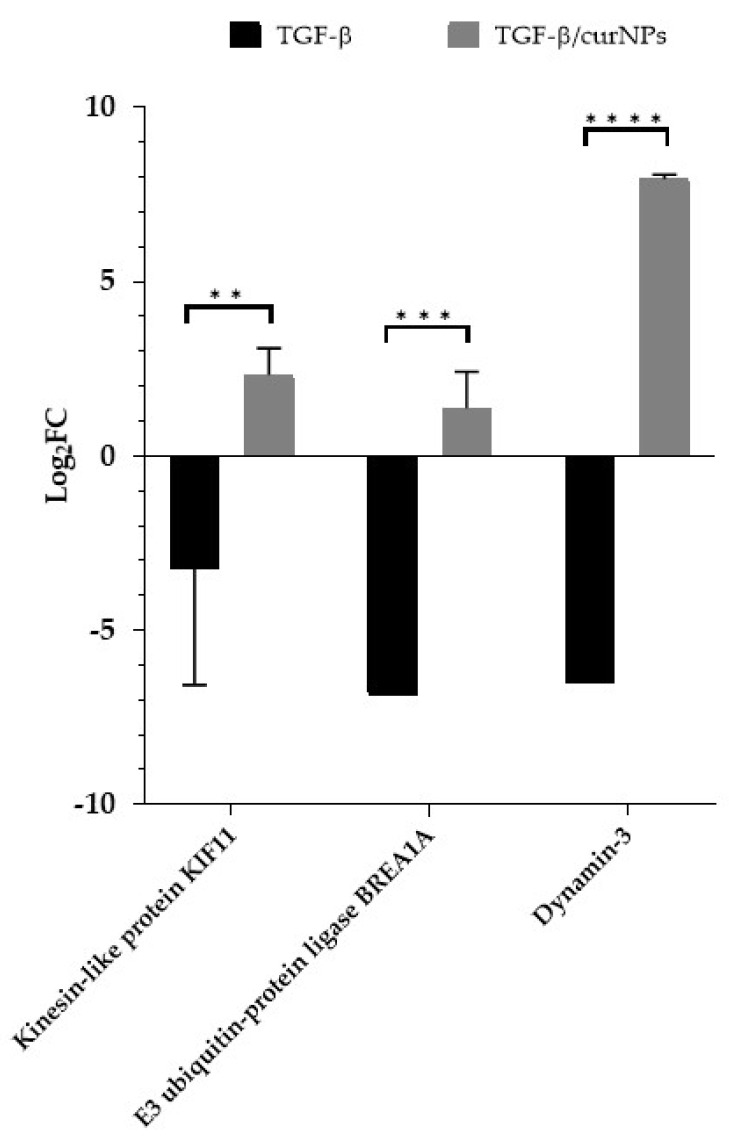
Expression levels, reported as log_2_(FC), of proteins deregulated by TGF-β and modulated by treatment with 10 µM CurNPs. Statistical analysis was performed with two-way ANOVA and Sidak’s multiple comparison test; ** *p* ≤ 0.01, *** *p* ≤ 0.001, **** *p* ≤ 0.0001.

In order to understand if some deregulated proteins in TGF-β-treated cells were also modulated by curNPs treatment, a comparative analysis was performed. Surprisingly, only three proteins (E3 ubiquitin-protein ligase BRE1A, kinesin-like protein KIF11, and dynamin-3) were shown to be common between the treatments (Table 3). As shown in Figure 4, the protein expression showed opposite trends. Indeed, these proteins were up-regulated during TGF-β/CurNPs treatment while they were down-regulated with TGF-β alone, showing a significant variation between the two groups. 

## 3. Discussion

Nutraceuticals play an important role in the management of multiple pathological conditions [21]. Among them, curcumin has been demonstrated with several properties, including antioxidant, anti-inflammatory, and anti-proliferative activity inhibiting multiple targets and molecular pathways [20]. However, the therapeutic use of curcumin is strongly limited by its low water solubility, which impairs its bioavailability. Currently, nanomedicine has overcome these obstacles improving curcumin pharmacokinetics, efficacy, and cellular uptake. In recent years, the anti-carcinogenic and anti-fibrotic activities of curNPs have been investigated in the hepatic field [6,7,8,9]; however, no study has evaluated its potentiality in the fibrotic pathological conditions that affect cholangiocytes. 

**Figure 5:**
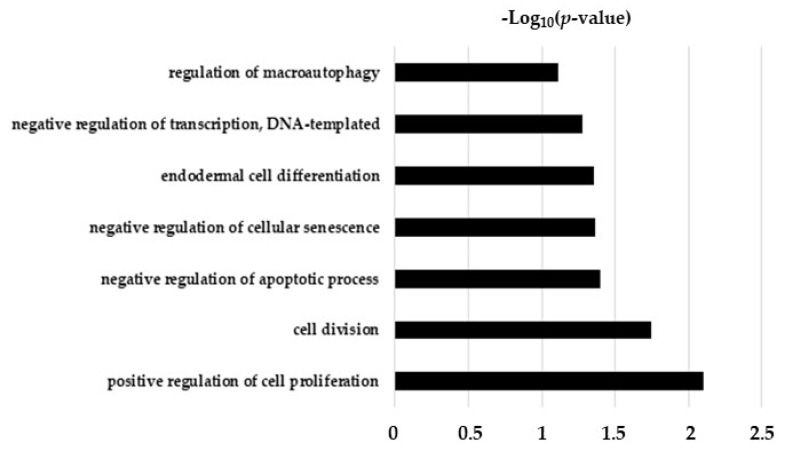
Biological function analysis of differentially expressed proteins in TGF-β-treated cholangiocytes compared to the control cells.

In the present study, we synthesized, characterized, and identified a phototherapy formulation that was CurNPs-based (Supplementary Materials, Figures S1 and S2). Following the cytotoxicity assay (Figure 1), 10 µM CurNPs was selected as the best concentration to investigate the potential effects of curcumin in a well-established in vitro model of TGFβ-damaged cholangiocytes, applying a SWATH-MS-based quantitative proteomic approach [18]. In TGFβ-treated cholangiocytes, we highlighted the up-regulation of several proteins directly involved in the cell cycle, such as the Bcl-2-like protein 12, centromere protein F, and Proline Rich Coiled-Coil 1 protein (PRRC1) (Table 1). Moreover, palladin, cullin-3, tensin-1, and moesin, which interacted with actin filaments, were also up-regulated following TGF-β treatment (Table 1). The role of the TGF-β family in controlling many aspects of cellular behavior, including proliferation, apoptosis, dormancy, autophagy, and senescence, is well known [11,22]. According to our data, the enriched biological processes of deregulated proteins in TGF-β-treated cholangiocytes were primarily related to the cell cycle, especially to cell proliferation and mitosis (Figure 5). Thus, our proteomic analysis, together with the analysis of biological processes, seemed to support the idea that TGF-β perturbed the cholangiocytes’ choice between proliferation, cell-cycle arrest, and apoptosis, promoting cell survival and proliferation. 

In addition, TGF-β induced a more than 290-fold increase in the fibronectin level compared to the control cells, which could be related to the well-documented pro-fibrotic properties of this cytokine [23]. Globally, our data indicated the pro-inflammatory and pro-fibrotic role of TGF-β in human cholangiocytes, together with its ability to perturb the cell cycle and related cytoskeleton dynamic.

In our proteomic analysis, we also identified cornifin-A as a marked down-regulated protein (FC = 0.25) compared to the control cells. Cornifin-A is part of a small proline-rich protein (SPRR) and was recently identified in the biliary epithelium. Demetris and colleagues reported that SPRR2A overexpression within in vitro biliary epithelial cells increased resistance to oxidative injury and promoted wound restitution by enhancing the migration and acquisition of mesenchymal characteristics [24]. Similarly, Nozaki and colleagues showed increased SPRR2 expression in the biliary epithelium of mice after bile duct ligation: a surgical method used to induce liver fibrosis [25]. Although these sporadic data could suggest the decisive role of SPPR2A in the function of the biliary barrier, no data elucidated the role of cornifin-A (SPPR1A). TGF-β could directly interfere with the function of the biliary barrier through the mediation of cornifin-A, but further investigations were necessary to elucidate this aspect.

**Figure 6:**
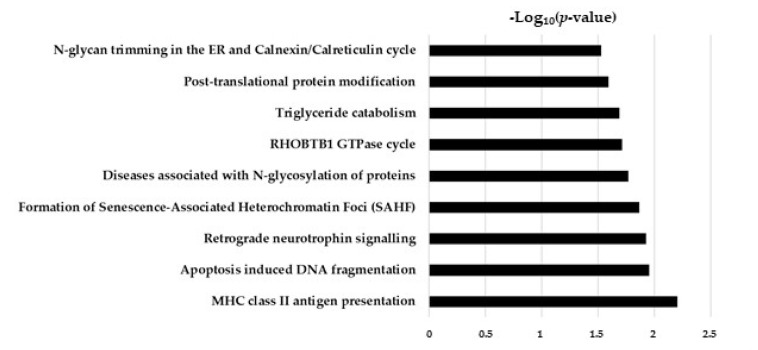
Pathway analysis of differentially expressed proteins in TGF-β/CurNPs-treated cholangiocytes.

The global proteome analysis of TGF-β/CurNPs-treated cholangiocytes highlighted interesting information regarding curNPs activity. As reported in Figure 6, these nutraceutic-modulated different pathways included antigen presentation, cell cycle, triglyceride metabolism, and post-translational protein modifications. 

CurNPs induced the up-regulation of histone H1.2 (FC = 2.94), which plays several important functions in multiple cellular processes, including apoptosis, autophagy, senescence, cell cycle control, and gene transcription, regulating chromatin dynamics [26,27]. According to the literature, our results could indicate the protective role of CurNPs through the induction of programmed cholangiocyte death, which acts as a barrier to further cellular damage by suppressing the growth of TGF-β-stressed cells.

In our previous work, we reported the impairment of Ca^2+^ homeostasis in human cholangiocytes under TGF-β treatment through the downregulation of multiple associated proteins such as calumin, calcium/calmodulin-dependent protein kinase type II subunit delta, caveolin-1, and NipSnap1 [18]. Human cholangiocytes have primary cilia with sensory functions that are linked to numerous receptors and ion-conducting channels and are involved in several cellular mechanisms, including calcium regulation and bile secretion [28,29,30,31]. Interestingly, in the present study, CurNPs determined the marked up-regulation of three proteins (kinesin-like protein KIF11, thioredoxin domain-containing protein 15, and dynamin-3) which were part of a primary cilium structure and the functions of most eukaryotic cells and probably also cholangiocytes. In human retinal pigment epithelial cells, the siRNA knockdown of the thioredoxin domain-containing protein 15 exerted alterations in ciliogenesis, with a significant reduction in ciliated cells compared to the control cells [32]. The kinesin-like protein KIF11 is primarily known as a mitotic regulator [33]; however, a non-mitotic role has recently emerged, suggesting its association with the primary cilia [34]. In that study, Zalenski and colleagues demonstrated how the kinesin-like protein KIF11 was localized in the basal bodies of primary cilia in multiple cell types, regulating cilia dynamics. Moreover, decreased protein levels were correlated to an increase in the number of ciliated cells, while the depletion of kinesin-like protein KIF11 determined a modification in the kinetics of cilia disassembly and increased the cilium length. In primary sclerosing cholangitis (PSC), which is a chronic and progressive cholestatic disease, cholangiocytes showed longer primary cilia compared to normal cholangiocytes, and this phenomenon impaired their physiological functions [35]. Although the thioredoxin domain-containing protein 15 expression was not perturbed by TGF-β, kinesin-like protein KIF11 levels were greatly reduced compared to the control cells (Figure 4), suggesting the possible role of TGF-β in the deregulation of cholangiocytes’ cilia function. Interestingly, CurNPs treatment reverted the effect of TGF-β on kinesin-like protein KIF11 expression (Figure 4) and also promoted increased levels of thioredoxin domain-containing protein 15 (Table 2). These data support the idea that CurNPs could restore the physiological function of cilia epithelium that was impaired in TGF-β-stressed cholangiocytes. In many cell types, the proximal part of the primary cilium presented an invagination of the plasma membrane, also known as a ciliary pocket, which is a specialized site for endocytosis [36]. In TGF-β-treated cholangiocytes, we reported a marked reduction in dynamin-3 levels (Table 1), which is one of the major proteins involved in endocytosis [37]. Recently, Inokawa and colleagues reported the decreased expression of dynamin-3 in hepatocellular carcinoma tissue [38]. Moreover, patients with reduced levels of this protein in these tumor tissues exhibit worse prognoses compared to patients without a decreased expression. Our results demonstrated that CurNPs reverted the effect of increasing TGF-β in the dynamin-3 levels of human cholangiocytes (Figure 4). These data suggest that nutraceutic positively modulates the endocytic processes that would be compromised in TGF-β-treated cholangiocytes, restoring the dynamin-3 levels whose reduction is also correlated with malignancy. 

Considering the well-documented anti-inflammatory properties of curcumin in liver diseases [19], in the present study, we investigated this effect in TGF-β-treated cholangiocytes. As reported in Figure 2, CurNPs significantly reduced the level of IL-6 that was increased following TGF-β treatment. Interestingly, our proteomic analysis highlighted the downregulation of E3 ubiquitin-protein ligase BRE1A in TGF-β-treated cholangiocytes and an opposite trend following CurNPs treatment (Figure 4). Recent findings correlated the overexpression of E3 ubiquitin-protein ligase BRE1A with the reversion of the TGF-β-induced activation of fibrotic proteins (VEGFA, TNF-α, and IL-6) in a human hepatic stellate cell line LX-2 [39]. According to these findings, our data could indicate the potential anti-fibrotic activity of CurNPs by increasing E3 ubiquitin-protein ligase BRE1A levels and reducing fibrotic mediators (i.e., IL-6).

**Figure 7:**
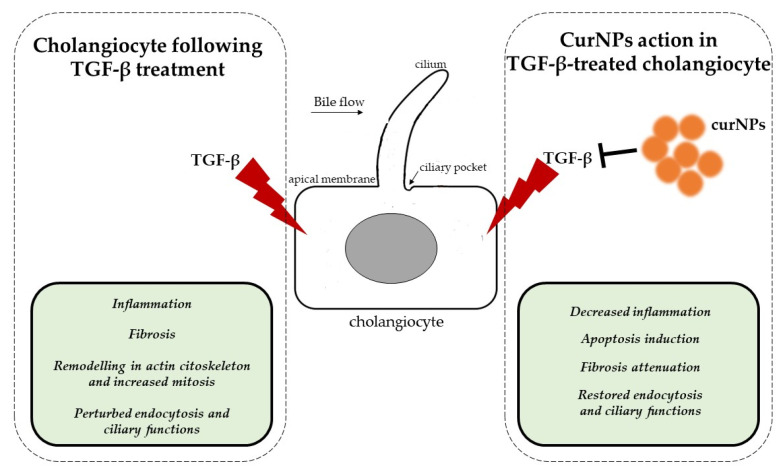
Representative image of the possible protective role of CurNPs in TGF-β-treated cholangiocytes.

In conclusion, we highlighted several aspects of curNPs activity on human cholangiocytes whose functions were perturbed during TGF-β stress, as reported in Figure 7. 

In particular, we confirmed the anti-inflammatory properties of this nutraceutic and the induction of programmed cell death to counteract the cellular damages induced by TGF-β. In human cholangiocytes, this cytokine is also responsible for the fibrotic processes, and CurNPs could attenuate this condition by reverting the expression of key mediators. In our study, TGF-β seemed to perturbate the cholangiocyte cilia functions and endocytic processes that could be restored by CurNPs. Given the crucial importance of deregulated cholangiocyte functions during cholangiopathies [40], our results lay the basis for a better understanding of the pathophysiology of cholangiocytes and could represent a rationale for the development of more targeted therapies.

## 4. Materials and Methods

### 4.1. Materials 

Primary human cholangiocytes (catalog number: 36755-12) and their specific growth medium without serum were purchased from Celprogen (Torrance, CA, USA). Curcumin (Merck, Rahway, NJ, USA), dimethyl sulfoxide (Merck, Rahway, NJ, USA), Ethanol absolute (Merck, Rahway, NJ, USA), fetal bovine serum (FBS, Gibco^®^, Carlsbad, CA, USA), Dulbecco’s Modified Eagle Medium (DMEM, Gibco^®^, Grand Island, NY, USA), Penicillin-Streptomycin (Gibco^®^, Grand Island, NY, USA), Trypsin-EDTA (Gibco^®^, Grand Island, NY, USA), Sirius Red/Fast Green Staining Collagen (Chondrex, Redmond, WA, USA), fertilized chicken eggs (PT. Lestari Farm, Sragen, Indonesia), distilled water (IPHA Laboratories, Bandung, Indonesia), ethyl acetate (Brataco, Jakarta, Indonesia), ascorbic acid, PBS pH 7.4, and 1,1-diphenyl-2-picrylhydrazyl (DPPH) were purchased from Sigma-Aldrich (St. Louis, MO, USA). TGF-β (T7039) was purchased from Merck. Formic acid (5.33002), trifluoroacetic acid (80457), and ammonium bicarbonate (Ambic, 40867), all eluent additives for LC-MS, were purchased, respectively, from Merck (Darmstadt, Germany), Sigma-Aldrich (St. Louis, MO, USA) and Fluka Analytical (Sigma-Aldrich, St. Louis, MO, USA). Acetonitrile (1.00029) hypergrade solvent LiChrosolv for LC-MS was bought from Merck (Darmstadt, Germany), while sodium deoxycholate (D6750) and albumin from bovine serum (A7906) from Sigma-Aldrich (St. Louis, MO, USA). Reagent A (23228) and reagent B (1859078) for Pierce BCA (bicinchoninic acid) Protein Assay were purchased from Thermo Scientific (Rockford, IL, USA). Iodoacetamide (RPN6302V), dithiothreitol (D1 309.0010), and trypsin modified sequencing grade (11418033001) were obtained, respectively, from GE Healthcare (Chicago, IL, USA), Duchefa Biochemie (Haarlem, The Netherlands) and Roche (Indianapolis, IN, USA). Mobicol spin columns (M1003) and their filters (M2110) 10 µm pore size were purchased from Mo Bi Tec (Goettingen, Germany), while VersaFlash spherical C18 bonded flash silica from Supelco Analytical (Bellefonte, PA, USA). Milli-Q deionized water was filtered on a Millipak filter (0.22 µm, MPGL040001) and purified on an LC-Pak cartridge (C18, LCPAK0001); all Millipore, Bedford, MA, USA). Beta-galactosidase digested (4333606) was obtained from AB Sciex (Framingham, MA, USA).

### 4.2. CurNPs Synthesis

CurNPs were prepared by nanoprecipitation in an Asia Syrris microfluidic setup (Syrris, Royston, UK). Briefly, a Cur solution in DMSO (10 mM) was mixed with deionized water at 1:158, 1:316, and 1:625 volume ratios using a 250 μL microfluidic mixer chip. The total flow rate was maintained at 50 μL/min, and the temperature was controlled at 25 °C. An eluted suspension was collected and stored at 4 °C until analysis. DLS analyses were performed on a Malvern Zetasizer ZS90 dynamic light scattering, using default settings. Nanoparticles were stable for more than 80 days without appreciable signs of degradation, as indicated by the DLS analyses performed on aged samples.

### 4.3. CurNPs Stability in Aqueous Media

The concentration and the stability of CurNPs, once added to the PBS or culture medium (with different percentages of FBS), were evaluated by UV-VIS spectrophotometric and spectrofluorimetric analysis performed on a Tecan Infinite M200 PRO plate reader, using Corning® 96 Well Clear Flat Bottom UV-Transparent Microplates and 200 μL of each solution.

### 4.4. CurNPs Cytotoxicity Assay

In total, 2000 human cholangiocytes per well were seeded in 96-well cell culture plates and incubated in a humidified atmosphere with 5% CO_2_ for 24 h. The culture medium was then replaced with CurNPs stock dispersion diluted in a medium supplemented with 1% FBS to achieve a final concentration of 50 µM, 35 µM, 20 µM, 10 µM, 5 µM and 1 µM. The cells were treated for 24 h and 48 h. Finally, 10 μL of the CCK-8 reagent (Sigma-Aldrich, St. Louis, MO, USA) was added into each well, and OD at 450 nm was measured using a microplate reader (FLUOstar Omega, BMG LABTECH, Ortenberg, Germany) after 2 h incubation at 37 °C.

### 4.5. Sample Preparation for Mass Spectrometry-Based Proteomics

The cell pellets were treated with 1% sodium deoxycholate in Ambic 50 mM, were sonicated for 5 min (five cycles of 20 s with an interval between cycles of 40 s on ice), and then clarified by centrifugation at 16,000× *g* for 10 min at 4 °C. The bicinchoninic acid assay was used to determine the protein concentration by using serum albumin as a standard. For each sample, 100 µg of protein was reduced with dithiothreitol (10 mM, for 30 min at 65 °C) and alkylated using iodoacetamide (20 mM, 30 min at 37 °C in dark conditions). Protein digestion was performed using trypsin (*w*/*w* ratio 1:50) at 37 °C for 18 h. The samples were incubated with 10% trifluoroacetic acid for 10 min at 37 °C to quench the trypsin reaction and remove sodium deoxycholate by acid precipitation. The samples were centrifuged at 16,000× g for 10 min and subsequently desalted with Mobicol spin columns equipped with 10 µm pore size filters and filled with a C18 stationary phase. The peptide mixtures were evaporated to dryness under a vacuum at 36 °C (Speed Vac Concentrator, Savant Instruments Inc., Farmingdale, NY, USA) before being resuspended in 50 µL of 5/95 CH_3_CN/0.1% HCOOH to achieve a final peptide concentration of 2 µg/µL and were finally transferred in plastic vials for LC-MS/MS analysis.

### 4.6. LC-MS/MS Analysis

Sample analyses were performed using a micro-HPLC (Eksigent Ekspert microLC 200) combined with a Triple TOF 5600 mass spectrometer equipped with a Turbo Ion Spray probe as the ion source (both AB Sciex, Concord, ON, Canada). Five µL of each sample/pool, set in an autosampler at 8 °C, was injected onto a C18 Jupiter column (150 mm × 0.3 mm i.d., 4 µm particle size, 90 Å), thermostatted at 30 °C and equipped with a micro trap C18 (10 mm × 0.3 mm) (both Phenomenex, Torrance, CA, USA). The flow rate was set at 5 µL/min, and the mobile phases A and B were, respectively, H_2_O and CHCN_3_, both with 0.1% HCOOH. The elution program was: 0 min, 5% B; 1 min, 5% B; 51 min, 22% B; 51.5 min, 90% B; 53.5 min, 90% B; 54 min, 5% B; 60 min, 5%B. Chromatographic performances and TOF accuracy were evaluated using an intra-run injection (5 µL) of beta-galactosidase 100 fmol/µL. The mass spectrometer was set in the positive ion mode, and the operation conditions of the ion source were the following: ion spray voltage floating 5.5 kV, probe temperature 150 °C, curtain gas 25 psi, ion source gas 1 and gas 2, respectively 30 and 20 psi, declustering potential 100 V. N_2_, as an inert gas, was used for CID (Collision Induced Dissociation) experiments.

A sample pool was analyzed (double injection) with an information-dependent acquisition (IDA) tandem mass spectrometry method based on an MS1 survey scan from which the 20 most abundant precursor ions were selected for subsequent CID fragmentation. The MS1 survey and MS2 scans were acquired over a mass range of 250–1250 *m*/*z* and 100–1500 *m*/*z*, with an accumulation time of 250 and 100 milliseconds, respectively. CID experiments were carried out using rolling collision energy that was automatically calculated according to the *m*/*z* and the charge in of the candidate ion, with a collision energy spread (CES) of 5 V. Precursor ions with a charge state from 2^+^ to 5^+^ were chosen to trigger the MS/MS experiments. The isolation width for precursor ion selection was set at 0.7 *m*/*z* on the Q1.

IDA results were used to generate the SWATH data-independent acquisition (DIA) method for protein quantitation. The SWATH acquisitions for each sample were performed over 50 overlapping isolation mass windows of variable length (minimum window width 3 Da, window overlap 1 Da) depending on the peptide density distribution along the entire mass range of 250–1250 *m*/*z*. Precursor ion activation was performed by CID as described before; however, an acquisition mass range of 230–1500 *m*/*z* and a charge state of 2^+^ were chosen. An accumulation time of 100 milliseconds for MS1 and 50 milliseconds for MS2 scans resulted in an overall duty cycle of 2.6 s (~15 points per elution peak).

### 4.7. IL-6 Dosage

According to the manufacturer’s protocol, IL-6 levels were determined using the Roche Elecsys Kit (09015604190). In total, 200 µL of the culture media underwent the first incubation with IL-6 specific antibodies, followed by a second incubation with IL-6 specific antibodies labeled with ruthenium complexes. After that, complexes were magnetically captured, and an electric voltage application determined the chemiluminescent emission that was directly proportional to the IL-6 concentration. 

### 4.8. Data Processing and Analysis

SWATH data analysis was carried out with DIA-NN (https://github.com/vdemichev/DiaNN) version 1.8, the free universal software for DIA proteomics data processing by V. Demichev, M. Ralser, and K. S. Lilley (The Francis Crick Institute, Molecular Biology of Metabolism laboratory, London, UK and Department of Biochemistry and The Milner Therapeutics Institute, University of Cambridge, Cambridge, UK).

First, SWATH raw file.wiff were converted into .wiff.dia and then used to generate the spectral library utilizing the following parameters: FASTA digest for library-free search/library generation enabled; Human Swiss-Prot FASTA database of 20404 reviewed, non-redundant protein species (downloaded on 4 January 2023); deep learning-based spectra, RTs and IMs prediction enabled; protease = trypsin; missed cleavages = 2; maximum number of variable modifications = 3; N-term M excision, C carbamidomethylation as fixed modification and M oxidation as variable modification all enabled; peptide length range = 7–30; precursor charge range = 1–4; precursor *m*/*z* range = 250–1250; fragment ion *m*/*z* range = 230–1500; generate spectral library enabled; precursor FDR = 1%; mass accuracy was determined automatically (mass accuracy = 0, MS1 accuracy = 0); use isotopologues, MBR (match between run) and remove likely interferences all enabled; neural network classifier = single-pass mode; protein inference = genes; quantification strategy = any LC (high accuracy); cross-run normalization = RT-dependent; library generation = smart profiling; where not specified, the standard settings were used.

For the protein quantitation step on files.wiff.dia, we kept the same parameters used above for spectral library generation, with only these exceptions: in the spectral library, the report-lib.tsv file generated was selected in the previous step; generate the spectral library, FASTA digest for library-free search/library generation, and deep learning-based spectra, RTs, and IMs prediction were all disabled; quantities matrices were enabled.

The report-quantification.pg_matrix output file of DIA-NN, reporting the log_2_ transformed cross-run normalized protein areas (obtained as the sum of the intensity for the top 3 precursor ions identified at 1% FDR and cross-run normalized), was used for further data analysis.

For each protein, the FC value was calculated as the ratio between the mean expression in TGF-β-treated cells and the mean expression in the control cells; and the mean expression in TGF-β/CurNPs-treated cells and the mean expression in TGF-β-treated cells. Proteins were considered differentially expressed when FCs were higher than 2.5 (FC ≤ 1/2.5 or FC ≥ 2.5). The values between different conditions were analyzed by two-way ANOVA followed by post hoc multiple comparison tests using GraphPad Prism version 8.0.0 for Windows (San Diego, CA, USA), and a *p*-value ≤ 0.05 was considered statistically significant. Biological significance and pathway analysis were conducted using DAVID Bioinformatics Resources 6.8 and the Reactome database, respectively. 

## Figures and Tables

**Table 1:** List of proteins that displayed more than a ±2.5-FC in TGF-β-treated cholangiocytes following 48 h treatment. The table reports the degrees of variation (evaluated as fold change) compared to the control cells and *p*-value. * Although this protein did not respect the established cut-off for expression levels, it was nonetheless indicated for its biological relevance and its significant down-regulation compared to the control cells.

Protein Name	Protein ID	FC	*p*-Value
Fibronectin	P02751	295.85	0.0045
Cullin-3	Q13618	13.69	0.0233
Tensin-1	Q9HBL0	7.15	0.008
Haloacid dehalogenase-like hydrolase domain-containing protein 2	Q9H0R4	6.05	0.0055
Palladin	Q8WX93	5.03	0.001
Bcl-2-like protein 12	Q9HB09	4.77	0.0042
Protein MTSS 1	O43312	4.51	0.0026
TSC22 domain family protein 1	Q15714	4.16	0.0016
Cytospin-A	Q69YQ0	3.19	0.0167
Phosphatidylcholine translocator ABCB4	P21439	3.10	0.049
Centromere protein F	P49454	3.04	0.0069
Four and a half LIM domains protein 2	Q14192	3.01	0.0036
High mobility group protein HMGI-C	P52926	3.00	0.0074
Protein PRRC1	Q96M27	3.00	0.0102
Zinc finger protein 746	Q6NUN9	2.95	0.0376
Protein FAM114A2	Q9NRY5	2.94	0.0367
Keratin, type II cytoskeletal 1	P04264	2.90	0.0210
Fos-related antigen 1	P15407	2.85	0.0212
Calpain small subunit 1	P04632	2.66	0.0266
SH3 and PX domain-containing protein 2A	Q5TCZ1	2.65	0.0075
Transducin-like enhancer protein 3	Q04726	2.62	0.0089
Zinc finger protein Helios	Q9UKS7	2.60	0.0036
Tetratricopeptide repeat protein 24	A2A3L6	2.57	0.0036
Moesin	P26038	2.51	0.0003
Kinesin-like protein KIF11 *	P52732	0.41	0.0132
Protein HEXIM1	O94992	0.40	0.0228
E3 ubiquitin-protein ligase BRE1A	Q5VTR2	0.30	0.0001
Cornifin-A	P35321	0.25	0.0009
Dynamin-3	Q9UQ16	0.01	<0.0001

**Table 2:** List of proteins that displayed more than ±2.5-FC in TGF-β/CurNPs-treated cholangiocytes. The table reports the degrees of variation (evaluated as FC) compared to TGF-β-treated cells (48 h).

Protein Name	Protein ID	FC	*p*-Value
Dynamin-3	Q9UQ16	248.1	0.0371
Kinesin-like protein KIF11	P52732	4.60	0.0081
E3 ubiquitin-protein ligase BRE1A	Q5VTR2	3.93	0.0462
Histone H1.2	P16403	2.94	0.0062
Nucleobindin-1	Q02818	2.79	0.03
Mannosyl-oligosaccharide glucosidase	Q13724	2.75	0.0018
Thioredoxin domain-containing protein 15	Q96J42	2.69	0.0086
Bromodomain adjacent to zinc finger domain protein 1A	Q9NRL2	2.67	0.0485
Fatty acid-binding protein, adipocyte	P15090	0.012	0.0001
Ras-related protein Rab-15	P59190	0.0014	0.0002

**Table 3:** List of proteins in common between TGF-β and TGF-β/CurNPs-treated cholangiocytes. The table reports the degrees of variation (evaluated as FC) compared to the control and TGF-β for TGF-β-treated cells and TGF-β/CurNPs-treated cells, respectively.

Protein Name	Protein ID	FC	
		TGF-β	TGF-β/CurNPs
E3 ubiquitin-protein ligase BRE1A	Q5VTR2	0.30	3.93
Kinesin-like protein KIF11	P52732	0.41	4.60
Dynamin-3	Q9UQ16	0.01	248.1

## Data Availability

The raw data supporting the conclusions of this article will be made available by the authors, without undue reservation.

## Supplementary Materials

The following supporting information can be downloaded at: https://www.mdpi.com/article/10.3390/ijms241310481/s1.
