# Supplementary material for: Proteomic Modulation in TGF-β-Treated Cholangiocytes Induced by Curcumin Nanoparticles
Source: Int J Mol Sci. 2023 Jun 22;24(13):10481. doi: 10.3390/ijms241310481 (PMC10341614; doi:10.3390/ijms241310481)
Supplement: Supplementary file 1 [file ijms-24-10481-s001.zip › ijms-2415462-supplementary.pdf]

Supplementary Materials

Figure S1: Dynamic light scattering analyses for different curNPs formulations.

Dynamic light scattering measurements for different curNPs formulations: curNPa (blue), curNPb (orange), curNPc (grey), curNPd (yellow) and curNPe (turquoise). DMSO/water volume ratios: 1:79 v/v (CurNPa), 1:158 v/v (CurNPb), 1:316 v/v (CurNPc), 1:625 v/v (CurNPd), and 1:1280 v/v (CurNPe).

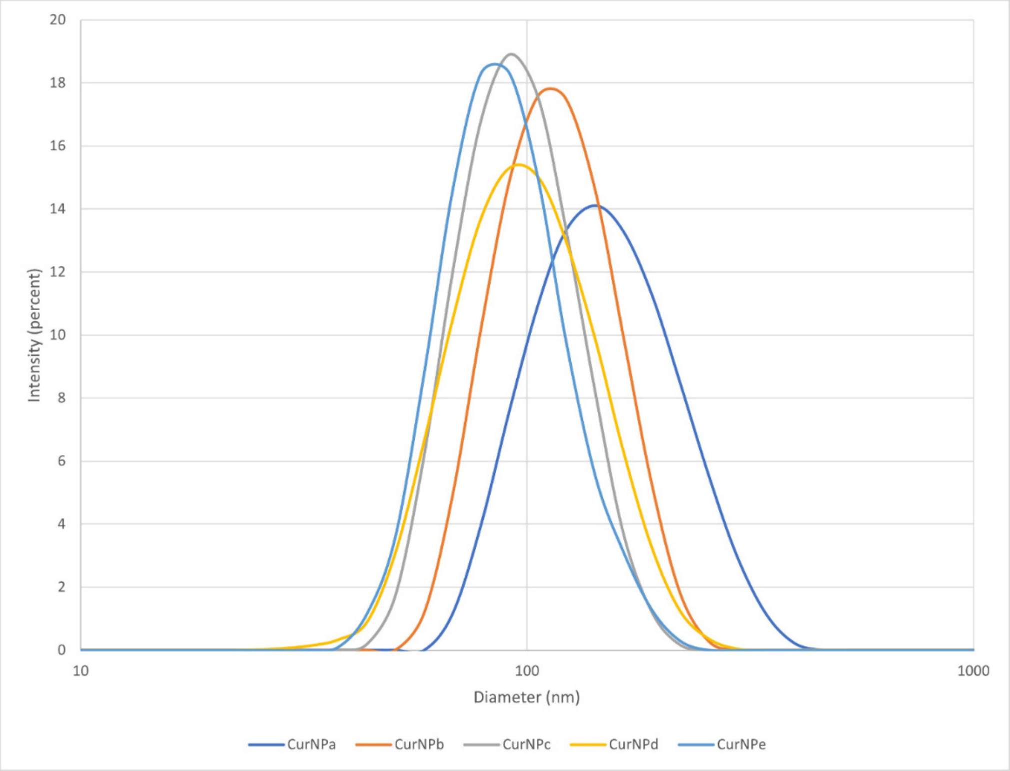

Figure S2: Curcumin and curNPs stability in aqueous media

Absorbance **(a)** and fluorescence **(b)** spectra (Ex 420 nm – gain 100) of curcumin solutions in DMSO (blank pure DMSO subtracted, Conc [uM]). Absorbance at 430 nm **(c)** and emitted Fluorescence at 540 nm **(d)** at t0 and t= 48 h of curcumin solutions in DMSO at different time points (t0= 1 h, t1= 18 h, t2= 24, t3= 42, t4= 48 h from sample preparation). Absorbance of curcumin solutions in DMSO is stable over time, while emitted fluorescence decreases. Effect of FBS on curcumin solubility evaluated by Absorbance (430 nm) **(e)** and Fluorescence **(f)** (540 nm (ex 420 nm, Gain 100). Curcumin solutions in PBS and in DMSO were analysed in panel **(g)** and **(h)**, respectively, with different percentage of FBS, following 30 min and 20h of incubation at 37°C. Different colour and discolouring over time (20 h) were reported in an aqueous medium. **(i)** Percentage of fluorescence for a dispersion 20 μM CuNPs in PBS, PBS with 1%, 2% and 10% FBS.

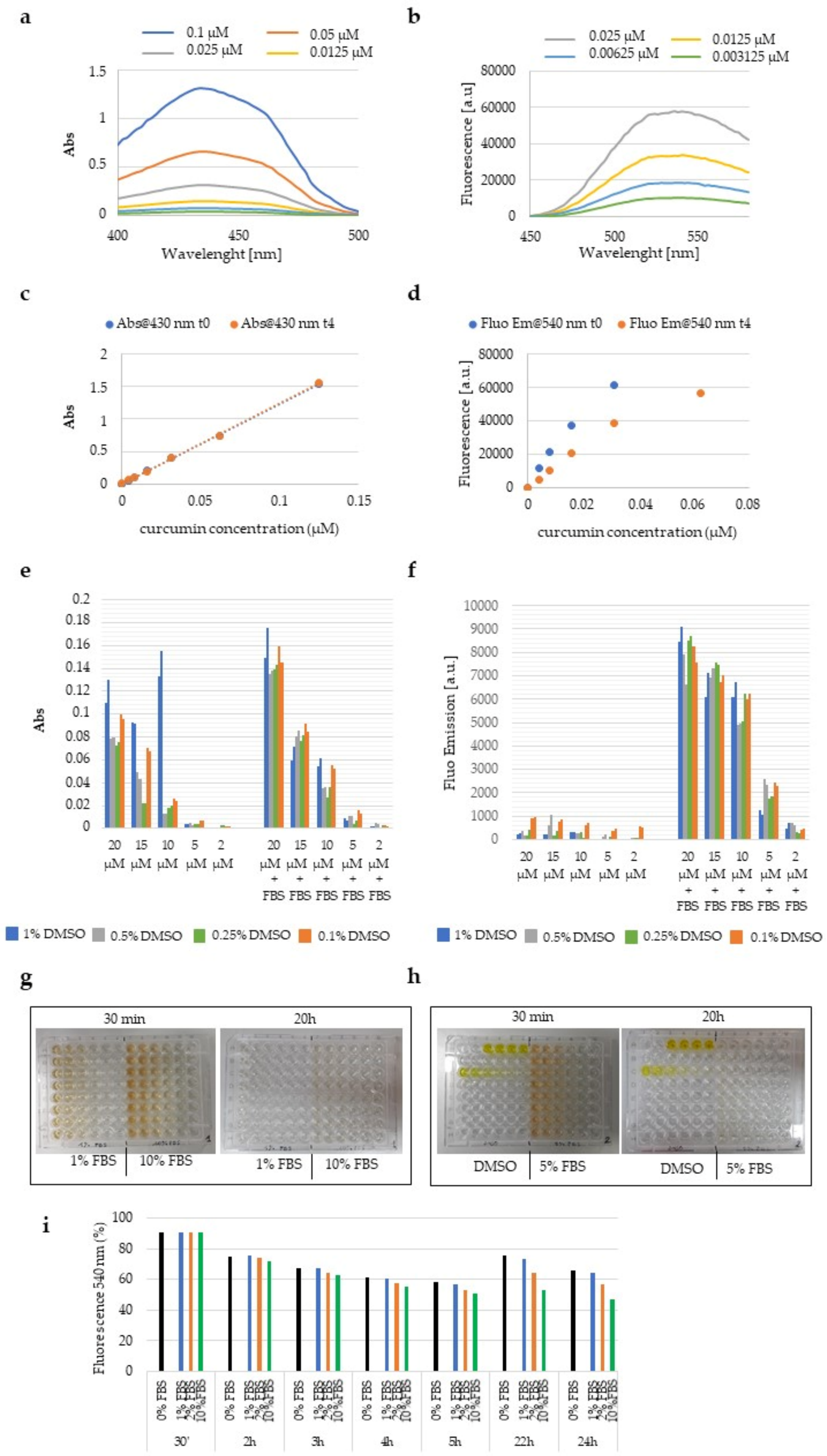
a
0.1 µM
0.05 µM
0.025 µM
0.0125 µM
Abs
Wavelenght [nm]
b
0.025 µM
0.0125 µM
0.00625 µM
0.003125 µM
Fluorescence [a.u]
Wavelenght [nm]
c
Abs@430 nm t0
Abs@430 nm t4
Abs
curcumin concentration (µM)
d
Fluo Em@540 nm t0
Fluo Em@540 nm t4
Fluorescence [a.u.]
curcumin concentration (µM)
e
Abs
20 µM
15 µM
10 µM
5 µM
2 µM
20 µM + FBS
15 µM + FBS
10 µM + FBS
5 µM + FBS
2 µM + FBS
1% DMSO
0.5% DMSO
0.25% DMSO
0.1% DMSO
f
Fluo Emission [a.u.]
20 µM
15 µM
10 µM
5 µM
2 µM
20 µM + FBS
15 µM + FBS
10 µM + FBS
5 µM + FBS
2 µM + FBS
1% DMSO
0.5% DMSO
0.25% DMSO
0.1% DMSO
g
30 min
20h
1% FBS
10% FBS
h
30 min
20h
DMSO
5% FBS
i
Fluorescence 540 nm (%)
0% FBS
1% FBS
5% FBS
10% FBS
30'
2h
3h
4h
5h
22h
24h
